# Impact of age on the use of adjuvant treatments in patients undergoing surgery for colorectal cancer: patients with stage III colon or stage II/III rectal cancer

**DOI:** 10.1186/s12885-019-5910-z

**Published:** 2019-07-25

**Authors:** C. Sarasqueta, A. Perales, A. Escobar, M. Baré, M. Redondo, N. Fernández de Larrea, E. Briones, J. M. Piera, M. V. Zunzunegui, J. M. Quintana, the REDISECC-CARESS/CCR group, Jose María Quintana, Marisa Baré, Maximino Redondo, Eduardo Briones Pérez de la Briones, Nerea Fernández de Larrea, Cristina Sarasqueta, Antonio Escobar, Francisco Rivas, Maria Morales-Suárez, Juan Antonio Blasco, Isabel del Cura, Inmaculada Arostegui, Amaia Bilbao, Nerea González, Susana García-Gutiérrez, Iratxe Lafuente, Urko Aguirre, Miren Orive, Josune Martin, Ane Antón-Ladislao, Núria Torà, Marina Pont, María Purificación Martínez del, Alberto Loizate, Ignacio Zabalza, José Errasti, Antonio Gimeno, Santiago Lázaro, Mercè Comas, Jose María Enríquez-Navascues, Carlos Placer, Amaia Perales, Iñaki Urkidi, Jose María Erro, Enrique Cormenzana, Adelaida Lacasta, Pep Piera, Elena Campano, Ana Isabel Sotelo, Segundo Gómez-Abril, F. Medina-Cano, Julia Alcaide, Arturo Del Rey-Moreno, Manuel Jesús Alcántara, Rafael Campo, Alex Casalots, Carles Pericay, Maria José Gil, Miquel Pera, Pablo Collera, Josep Alfons Espinàs, Mercedes Martínez, Mireia Espallargues, Caridad Almazán, Paula Dujovne, José María Fernández-Cebrián, Rocío Anula, Julio Mayol, Ramón Cantero, Héctor Guadalajara, María Alexandra Heras, Damián García, Mariel Morey, Alberto Colina

**Affiliations:** 1 Biodonostia Health Research Institute - Donostia University Hospital / Red de Investigación en Servicios de Salud en Enfermedades Crónicas (REDISSEC), Paseo Dr. Beguiristain s/n, 20014 Donostia-San Sebastián, Gipuzkoa Spain; 2 Research Unit, Hospital Basurto / Red de Investigación en Servicios de Salud en Enfermedades Crónicas (REDISSEC), Avda Montevideo, 18, 48013 Bilbao, Bizkaia Spain; 3 Clinical Epidemiology and Cancer Screening, Corporació Sanitaria Parc Taulí / Red de Investigación en Servicios de Salud en Enfermedades Crónicas (REDISSEC), Parc Taulí 1, 08208 Sabadell, Barcelona, Spain; 4 Research Unit, Costa del Sol Hospital / Red de Investigación en Servicios de Salud en Enfermedades Crónicas (REDISSEC), Autovía A-7, Km 187, 29603 Marbella, Málaga, Spain; 5 Cancer and Environmental Epidemiology Unit, National Center for Epidemiology, Instituto de Salud Carlos III / Consortium for Biomedical Research in Epidemiology and Public Health (CIBERESP), Avda de Monforte de Lemos, 5, 28029 Madrid, Spain; 6 Epidemiology Unit, Seville Health District, Andalusian Health Service / Consortium for Biomedical Research in Epidemiology and Public Health (CIBERESP), Avda de la Constitución, 18, 41071 Seville, Spain; 7 Medical Oncology Unit, Donostia University Hospital, Paseo Dr. Beguiristain 109, 20014 Donostia-San Sebastián, Gipuzkoa Spain; 8 Departement de médecine sociale et préventive Institut de recherche en santé publique (IRSPUM), University of Montréal, Pavillon 7101, salle 3111 7101, Avenue du Parc Montréal, Montréal, Québec H3N 1X9 Canada; 9 Research Unit, Galdakao-Usansolo Hospital / REDISSEC, Labeaga Auzoa, 48960 Galdakao, Bizkaia Spain

**Keywords:** Colorectal cancer, Age, Equity, Adherence, Chemotherapy, Preoperative radiotherapy

## Abstract

**Background:**

Many older patients don’t receive appropriate oncological treatment. Our aim was to analyse whether there are age differences in the use of adjuvant chemotherapy and preoperative radiotherapy in patients with colorectal cancer.

**Methods:**

A prospective cohort study was conducted in 22 hospitals including 1157 patients with stage III colon or stage II/III rectal cancer who underwent surgery. Primary outcomes were the use of adjuvant chemotherapy for stage III colon cancer and preoperative radiotherapy for stage II/III rectal cancer. Generalised estimating equations were used to adjust for education, living arrangements, area deprivation, comorbidity and clinical tumour characteristics.

**Results:**

In colon cancer 92% of patients aged under 65 years, 77% of those aged 65 to 80 years and 27% of those aged over 80 years received adjuvant chemotherapy (χ^2^_trends_ < 0.001). In rectal cancer preoperative radiotherapy was used in 68% of patients aged under 65 years, 60% of those aged 65 to 80 years, and 42% of those aged over 80 years (χ^2^_trends_ < 0.001). Adjusting by comorbidity level, tumour characteristics and socioeconomic level, the odds ratio of use of chemotherapy compared with those under age 65, was 0.3 (0.1–0.6) and 0.04 (0.02–0.09) for those aged 65 to 80 and those aged over 80, respectively; similarly, the odds ratio of use of preoperative radiotherapy was 0.9 (0.6–1.4) and 0.5 (0.3–0.8) compared with those under 65 years of age.

**Conclusions:**

The probability of older patients with colorectal cancer receiving adjuvant chemotherapy and preoperative radiotherapy is lower than that of younger patients; many of them are not receiving the treatments recommended by clinical practice guidelines. Differences in comorbidity, tumour characteristics, curative resection, and socioeconomic factors do not explain this lower probability of treatment. Research is needed to identify the role of physical and cognitive functional status, doctors’ attitudes, and preferences of patients and their relatives, in the use of adjuvant therapies.

**Electronic supplementary material:**

The online version of this article (10.1186/s12885-019-5910-z) contains supplementary material, which is available to authorized users.

## Background

Evidence suggests that older patients can benefit from aggressive therapies as much as younger individuals can, improving their overall and disease-free survival [1]. Nevertheless, a high percentage of older patients do not receive standard cancer treatments [2–5]. A European study found that 69% of patients under 65 years old and only 16% of those over this age received adjuvant chemotherapy for stage III colon cancer [4]. Several authors have shown that these differences remain after adjusting for comorbidity [2, 6]. Age has also been associated with the frequency of use of radiotherapy [7–9]. In Sweden, preoperative radiotherapy for rectal cancer was given to 64% of patients under 65 years old, to 50% of 65 to 79 years old and to 15% of those 80 years of age or older [7]. In Canada, Eldin et al. observed that after adjusting for comorbidity and stage, age was the most important factor in determining the use of radiotherapy [9]. Most of the revised studies have reported results adjusting for comorbidity and stage, but studies are scarce that in addition have adjusted for the patient’s social position and living arrangements. None of the multicentre studies has taken into account the inter-hospital variability both in clinical practice and in hospital area’s material deprivation.

A greater toxicity of chemotherapy and radiotherapy in older patients with colorectal cancer might explain a lower adherence to clinical practice guidelines. Further, the exclusion of older patients from clinical trials means that there is limited scientific evidence concerning the efficacy and toxicity associated with treatments in this population. This has led to a lack of evidence-based clinical guidelines [3]. For tumours at some anatomical sites, radiation therapy has been found to be more toxic in patients of advanced ages, suggesting a need for closer monitoring [1]. Nevertheless, the majority of clinical trials including older patients with colorectal cancer have reported toxicity profiles similar to those observed in younger patients [10, 11]. In addition to these clinical factors, there are social factors that may place older patients at a disadvantage with respect to receiving treatments, such as having a lower socioeconomic level [12–14] and a lower level of education [15], as well as more frequently living alone [16].

The aims of this paper were a) to identify whether there are differences between age groups in the use of chemotherapy for stage III colon cancer and preoperative radiotherapy for stage II and III rectal cancer; and b) to assess whether these differences remain after adjusting for comorbidity, tumour characteristics, curative resection and social factors such as economic deprivation or living arrangements.

## Methods

**Fig. 1:**
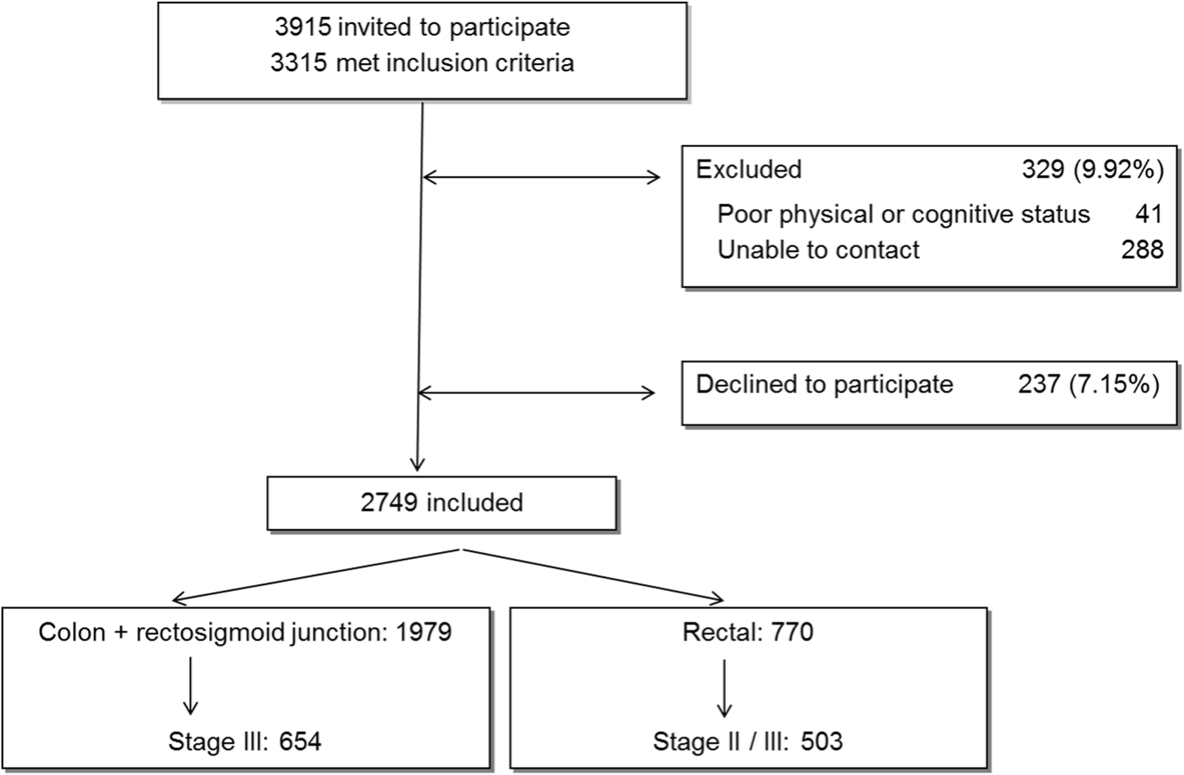
Flowchart of patients through the study and reasons for non-inclusion

Data were obtained by conducting a prospective multicentre cohort study in 22 hospitals in five autonomous regions in Spain. We included patients with primary invasive colon or rectal cancer who underwent programmed or urgent surgery between April 2010 and December 2012. A detailed protocol was published by Quintana et al. [17]. Among the 3315 patients who met the inclusion criteria, 41 were excluded from the study due to poor physical or cognitive status, and we failed to contact another 288. In addition, 237 (7.2%) declined to participate in the study (Fig. 1).

### Outcomes and covariates

The primary outcomes analysed were the use of adjuvant chemotherapy in stage III colon cancer and preoperative radiotherapy in stage II and III rectal cancer. Age was assessed at the time of diagnoses and arbitrarily categorized into three groups: younger (under 65 years of age), older (65 to 80 years) and oldest (over 80 years) patients.

We assessed prognostic factors, which according to the scientific literature, might be unevenly distributed between age groups: a) Social and economic variables: socioeconomic level, considering level of education and area of residence deprivation, which was calculated following the methodology of Esnaola et al. [18], for each census tract based on five 2001 census indicators related to occupation and educational attainment; living arrangements (alone or with others);

b) health behaviours: alcohol intake (greater than 80 g/day or not) and smoking habits (current smoker, ex-smoker, never smoker);

c) cancer family history and whether the diagnosis had been made through a screening programme or not;

d) health status: comorbidities, measured using the Charlson comorbidity index (CCI) [19], stratifying patients into three groups (0, 1, and 2 or more), and the American Society of Anesthesiologists (ASA) class [20], a proxy for the severity of patients’ comorbidities;

e) tumour characteristics: site (proximal colon, distal colon, rectosigmoid junction or rectum), histological findings (adenocarcinoma, mucinous adenocarcinoma, signet ring cell carcinoma, others), degree of differentiation (low, corresponding to tumours that are well or moderately well differentiated, or high, corresponding to poorly differentiated and undifferentiated tumours); h) tumour stage (according to the 7th edition of the TNM classification of the Union for International Cancer Control), assigning patients who underwent neoadjuvant treatment a clinical stage and those who underwent surgery as the first treatment a pathological stage, for statistical analysis;

f) surgery: profile of the surgeon (fully dedicated to coloproctology or not); type of surgery (elective/emergency); curative resection (no residual tumour (R0) or microscopic/macroscopic remnant of the tumour (R1/R2)); and finally whether a cancer committee was involved in the patient’s management, as a process indicator.

### Statistical analysis

First, potential prognostic factors were compared among the three age groups using Pearson chi-square test (χ^2^) and chi square test for trends (χ^2^_trends_). Then, the univariate association of each factor with the use of adjuvant chemotherapy and preoperative radiotherapy was investigated using Pearson chi square test for the categorical non ordinal variables and chi square test for trends for the ordinal variables. Multivariable analyses were performed with Generalised Estimating Equations, clustering by hospital, to assess the association between age and the use of chemotherapy and preoperative radiotherapy, adjusting for sociodemographic and clinical factors. This approach enabled us to construct multivariate models that take into account the correlation between individuals from the same hospital. An unstructured variance-covariance matrix was used. Potential confounders with *p* < 0.2 in the univariate analysis were entered simultaneously in the multivariable model using dummy variables. Missing data were imputed using the multiple imputation method available in SPSS which uses by default 5 iterations. The imputed variables were: level of education, deprivation index, screening, ASA class and alcohol intake. The variables used for the imputation were as follows: age, level of education, deprivation index, autonomous region, CCI, ASA class, alcohol intake and surgeon profile. The calculated measure of association was the odds ratio with the corresponding 95% confidence interval. Two-tailed tests were used, considering *p* values < 0.05 to be statistically significant. The analysis was performed using IBM SPSS, Statistics for Windows, v23, and Stata v14.

## Results

A total of 2749 patients were finally included in the study, among whom 654 had stage III colon cancer and 503 stage II or III rectal cancer (Fig. 1). This research report refers to these 1157 patients.

Patients included were significantly older than those who were excluded or not contactable (p, χ^2^ < 0.005), but differences with those who declined to participate were not statistically significant.

Of the included patients, 38.8% were under 65 years, 47.2% were between 65 and 80 years, and 13.9% were over 80 years of age. Approximately two thirds (65.2%) were men. Overall, 13% had not completed any formal education, and only 12% had university qualifications (short- or long-cycle degrees). Most participants (86%) lived with a relative.

Tables 1 and 2 indicate the observed differences between age groups, for colon and rectum respectively. Older patients were more likely to have a low education level (p, χ^2^_trends_ < 0.0005) and to live alone (p, χ^2^ < 0.0005). No significant differences were found in deprivation of the area of residence (*p* = 0.9). Younger patients were more likely to report a family history of cancer (p, χ^2^ < 0.05). The proportion of patients who have never smoked increases with age (p, χ^2^ < 0.05) and comorbidity increases with age (p, χ^2^_trends_ < 0.0005). In colon cancer there were no age significant differences in tumour sites, histological classification, degree of differentiation or, in rectal cancer, in stage at diagnosis. Finally, we did not find differences in curative resections (R0) by age.

**Table 1:** Distribution of social, health and clinical patient’s variables by age groups in stage III colon cancer (*n* = 654)

	N	< 65 years *N* = 246 n (%)	65–80 years *N* = 311 n (%)	> 80 years *N* = 97 n (%)	*P* value^a^
Sociodemographic variables					
Sex	654				
Male		151 (61.4)	205 (65.9)	60 (61.9)	0.50
Female		95 (38.6)	106 (34.1)	37 (38.1)	0.65
Deprivation index	624				
Quartile 1 least deprived		49 (21.2)	67 (22.3)	17 (18.5)	0.67
Quartile 2		64 (27.7)	101 (33.6)	31 (33.7)	0.78
Quartile 3		67 (29.0)	72 (23.9)	22 (23.9)	
Quartile 4 most deprived		51 (22.1)	61 (20.3)	22 (23.9)	
Level of education	534				
Illiterate or with no formal education		10 (4.8)	46 (18.2)	15 (20.5)	< 0.0005
Primary		123 (59.1)	162 (64.0)	50 (68.5)	< 0.0005
Secondary		35 (16.8)	24 (9.5)	3 (4.1)	
University		40 (19.2)	21 (8.3)	5 (6.8)	
Living arrangements	522				
Living alone		23 (11.5)	42 (17.0)	14 (18.7)	0.18
Living with others		177 (88.5)	205 (83.0)	61 (81.3)	0.08
Family history of cancer	584				
No		118 (52.2)	175 (63.9)	68 (81.0)	< 0.0005
Yes		108 (47.8)	99 (36.1)	16 (19.0)	< 0.0005
Screening	622				
No		174 (74.0)	250 (84.7)	86 (93.5)	< 0.0005
Yes		61 (26.0)	45 (15.3)	6 (6.5)	< 0.0005
Health behaviours and comorbidities					
Smoking habits	648				
Never smoker		112 (45.5)	154 (50.0)	50 (53.2)	0.01
Current smoker		42 (17.1)	31 (10.1)	4 (4.3)	0.79
Ex-smoker		92 (37.4)	123 (39.9)	40 (42.6)	
Alcohol	612				
No		188 (83.9)	257 (86.2)	84 (93.3)	0.09
Yes		36 (16.1)	41 (13.8)	6 (6.7)	0.04
ASA class	633				
I-II		175 (73.2)	157 (52.2)	30 (32.3)	< 0.0005
III		60 (25.1)	127 (42.2)	52 (55.9)	< 0.0005
IV		4 (1.7)	17 (5.6)	11 (11.8)	
Charlson Index	654				
0		163 (66.3)	158 (50.8)	40 (41.2)	< 0.0005
1		49 (19.9)	80 (25.7)	26 (26.8)	< 0.0005
≥ 2		34 (13.8)	73 (23.5)	31 (32.0)	
Tumour characteristics					
Site	654				
Rectosigmoid junction		40 (16.3)	45 (14.5)	11 (11.3)	0.72
Distal colon		108 (43.9)	140 (45.0)	41 (42.3)	0.21
Proximal colon		98 (39.8)	126 (40.5)	45 (46.4)	
Histological classification	643				
Adenocarcinoma		219 (91.3)	274 (89.3)	87 (90.6)	0.18
Mucinous adenocarcinoma		16 (6.7)	27 (8.8)	9 (9.4)	0.69
Signet-ring cell carcinoma		2 (0.8)	6 (2.0)	0 (0.0)	
Other types of carcinoma		3 (1.3)	0 (0.0)	0 (0.0)	
Degree of differentiation	573				
Low grade		165 (79.3)	226 (81.9)	79 (88.8)	0.15
High grade		43 (20.7)	50 (18.1)	10 (11.2)	0.07
Intervention					
Main intervention	654				
Elective		232 (94.3)	300 (96.5)	87 (89.7)	0.03
Emergency		14 (5.7)	11 (3.5)	10 (10.3)	0.32
Curative resection	618				
R0		218 (92.4)	261 (90.0)	84 (91.3)	0.63
R1 / R2		18 (7.6)	29 (10.0)	8 (8.7)	0.56
Surgeon’s profile	615				
General		61 (27.0)	96 (32.5)	36 (38.3)	0.12
Coloproctologist		165 (73.0)	199 (67.5)	58 (61.7)	0.04
Cancer committee	617				
No		77 (32.9)	127 (43.6)	44 (47.8)	0.01
Yes		157 (67.1)	164 (56.4)	48 (52.2)	0.004

^a^Pearson Chi-square test to generate upper *P* value and chi-square test for trends to generate lower *P* value

**Table 2:** Distribution of social, health and clinical patient’s variables by age groups in stage II, III rectal cancer (*n* = 503)^a^

	N	< 65 years *N* = 203 n (%)	65–80 years *N* = 235 n (%)	> 80 years *N* = 64 n (%)	*P* value^b^
Sociodemographic variables					
Sex	502				
Male		128 (63.1)	169 (71.9)	41 (64.1)	0.12
Female		75 (36.9)	66 (28.1)	23 (35.9)	0.35
Deprivation index	476				
Quartile 1 least deprived		28 (14.7)	41 (18.6)	12 (18.8)	0.66
Quartile 2		68 (35.6)	72 (32.6)	16 (25.0)	0.80
Quartile 3		57 (29.8)	71 (32.1)	24 (37.5)	
Quartile 4 most deprived		38 (19.9)	37 (16.7)	12 (18.8)	
Level of education	408				
Illiterate or with no formal education		9 (5.3)	30 (15.9)	11 (22.4)	< 0.0005
Primary		100 (58.8)	137 (72.5)	33 (67.3)	< 0.0005
Secondary		34 (20.0)	8 (4.2)	1 (2.0)	
University		27 (15.9)	14 (7.4)	4 (8.2)	
Living arrangements	403				
Living alone		11 (6.7)	30 (15.8)	9 (18.8)	0.01
Living with others		154 (93.3)	160 (84.2)	39 (81.3)	0.005
Family history of cancer	464				
No		97 (51.9)	126 (58.3)	42 (68.9)	0.06
Yes		90 (48.1)	90 (41.7)	19 (31.1)	0.02
Screening	478				
No		167 (87.0)	199 (88.8)	58 (93.5)	0.36
Yes		25 (13.0)	25 (11.2)	4 (6.5)	0.18
Health behaviours and comorbidities					
Smoking habits	498				
Never smoker		82 (40.4)	104 (44.8)	37 (58.7)	0.001
Current smoker		49 (24.1)	30 (12.9)	3 (4.8)	0.37
Ex-smoker		72 (35.5)	98 (42.2)	23 (36.5)	
Alcohol	483				
No		175 (89.3)	198 (87.2)	56 (93.3)	0.39
Yes		21 (10.7)	29 (12.8)	4 (6.7)	0.70
ASA class	490				
I-II		144 (73.1)	118 (51.5)	31 (48.4)	< 0.0005
III		51 (25.9)	101 (44.1)	31 (48.4)	< 0.0005
IV		2 (1.0)	10 (4.4)	2 (3.1)	
Charlson Index	502				
0		134 (66.0)	118 (50.2)	25 (39.1)	< 0.0005
1		43 (21.2)	60 (25.5)	18 (28.1)	< 0.0005
≥ 2		26 (12.8)	57 (24.3)	21 (32.8)	
Tumour characteristics					
Histological classification	467				
Adenocarcinoma		181 (96.8)	201 (92.6)	58 (92.1)	0.05
Mucinous adenocarcinoma		5 (2.7)	16 (7.4)	4 (6.3)	0.05
Signet-ring cell carcinoma		1 (0.5)	0 (0.0)	0 (0.0)	
Other types of carcinoma		0 (0.0)	0 (0.0)	1 (1.6)	
Degree of differentiation	389				
Low grade		137 (86.7)	153 (86.0)	48 (90.6)	0.68
High grade		21 (13.3)	25 (14.0)	5 (9.4)	0.62
Stage at diagnosis (pTNM or cTNM)	502				
II		61 (30.0)	76 (32.3)	27 (42.2)	0.19
III		142 (70.0)	159 (67.7)	37 (57.8)	0.11
Intervention					
Main intervention	502				
Elective		200 (98.5)	234 (99.6)	64 (100.0)	0.35
Emergency		3 (1.5)	1 (0.4)	0 (0.0)	0.16
Curative resection	476				
R0		162 (84.4)	193 (85.4)	52 (89.7)	0.60
R1/R2		30 (15.6)	33 (14.6)	6 (10.3)	0.37
Surgeon’s profile	476				
General		59 (30.3)	71 (32.3)	18 (29.5)	0.87
Coloproctologist		136 (69.7)	149 (67.7)	43 (70.5)	0.92
Cancer committee	418				
No		52 (27.2)	74 (33.2)	19 (29.7)	0.42
Yes		139 (72.8)	149 (66.8)	45 (70.3)	0.42

^a^Age was missing in a case

^b^Pearson Chi-square test to generate upper *P* value and chi-square test for trends to generate lower *P* value

Among the main differences in colon and rectal cancer, we highlight the following: younger patients were more likely to have undergone screening (p, χ^2^ < 0.0005) in colon cancer but there were no significant differences in rectal cancer; among those with colon cancer, patients over 80 years of age were more likely to have had emergency surgery (p, χ^2^ = 0.04) compared with those under age 80; with increasing age, the number of surgical interventions done by surgeons specialized in coloproctology decreased (p, χ^2^_trends_ = 0.04) and the proportion of cases reviewed by an interdisciplinary tumor committee decreased (p, χ^2^_trends_ = 0.004). These differences were not observed among those with rectal cancer.

Table S1 reports the frequencies of imputed variables before and after imputation. The distribution of the imputed values can be seen to be homogenous (Additional file 1: Table S1).

### Adjuvant chemotherapy for patients with colon cancer

**Fig. 2:**
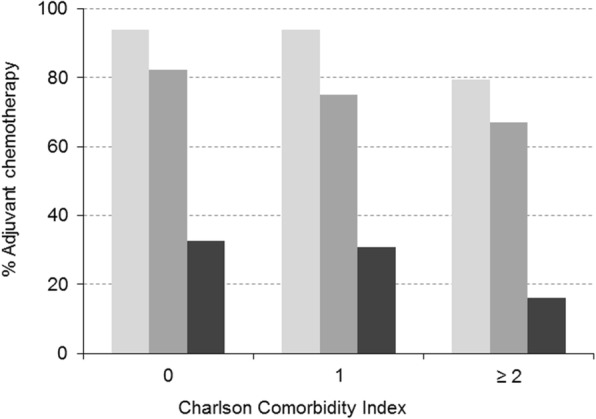
Percentage of patients with stage III colon cancer who received chemotherapy by age and number of comorbidities. Legend: Age (years)  < 65,  65–80,  > 80

Of the 654 patients with stage III colon or rectosigmoid cancer identified, 75% received chemotherapy after surgical resection. Table 3A summarises the univariate association of patient characteristics with chemotherapy. The use of this therapy decreased significantly with age, from 91.9% in the youngest age group to 76.7% in the older group to only 26.8% in the oldest patients (p, χ^2^_trends_ < 0.0005). No significant difference in use of adjuvant chemotherapy was observed by sex. A higher level of comorbidity was also associated with less use of chemotherapy, with a rate of 82% in patients with no comorbidities falling to just 58.7% in those with a CCI of 2 or more. Nevertheless, we should note that even among patients with no comorbidities, older age was also associated with less use of chemotherapy; the rates were 94, 82 and 33% for those under 65, between 65 and 80, and over 80 years of age, respectively (p, χ^2^_trends_ < 0.0005) (Fig. 2). Table 3B shows the multivariable results. There was a significant negative association between age and the use of chemotherapy after simultaneously adjusting for comorbidity, tumour characteristics (such as the site and degree of differentiation) and level of education. Compared to younger patients, the adjusted OR was 0.3 (95% CI: 0.1–0.6) for the older and 0.04 (95% CI: 0.02–0.09) for the oldest age groups. We found no significant association between chemotherapy use and either participation of the cancer committee in the management of the patient or the surgeon’s specialisation. The outcome of the surgery did not have a significant effect on the chemotherapy use.

**Table 3:** Crude and adjusted analysis of the association between age and adjuvant chemotherapy in stage III colon cancer

	Adjuvant chemotherapy for stage III colon cancer			
	3A. Univariate analysis		3B. Multivariate analysis	
	n (%)	*P* value	Odds Ratio (95% CI)	*P* value
Age, years				
< 65	226 (91.9)	< 0.0005^b^	1	
65–80	239 (76.8)		0.3 (0.1–0.6)	0.001
> 80	26 (26.8)		0.04 (0.02–0.09)	< 0.0005
Sex				
Male	309 (74.3)	0.57^a^		
Female	182 (76.5)			
Deprivation index				
Quartile 1	106 (79.7)	0.29^b^		
Quartile 2	142 (72.4)			
Quartile 3	121 (75.2)			
Quartile 4	97 (72.4)			
Level of education				
No formal	47 (66.2)	< 0.0005^b^	1	
Primary	253 (75.5)		1.2 (0.7–2.2)	0.46
Secondary	53 (85.5)		1.6 (0.4–5.5)	0.46
University	59 (89.4)		1.6 (0.5–5.7)	0.45
Living arrangement				
Living alone	56 (70.9)	0.2^a^		
Living with others	345 (77.9)			
Family history of cancer				
No	257 (71.2)	< 0.0005^a^	1	
Yes	194 (87.0)		1.9 (0.7–5.0)	0.21
Screening				
No	368 (72.2)	0.001^a^	1	
Yes	97 (86.6)		1.0 (0.4–2.2)	0.99
Smoking habits				
Never smoker	241 (76.3)	0.516^a^		
Current smoker	61 (79.2)			
Ex-smoker	187 (73.3)			
Alcohol				
No	392 (74.1)	0.42^a^		
Yes	63 (75.9)			
Charlson index				
0	296 (82.0)	< 0.0005^b^	1	
1	114 (73.5)		0.8 (0.5–1.4)	0.43
≥ 2	81 (58.7)		0.6 (0.3–1.2)	0.17
ASA class				
I-II	309 (85.4)	< 0.0005^b^	1	
III	155 (64.9)		0.6 (0.3–1.1)	0.10
IV	10 (31.3)		0.1 (0.03–0.3)	< 0.0005
Site				
Rectosigmoid junction	78 (81.3)	0.19^a^	1	
Distal colon	219 (75.8)		0.5 (0.2–1.4)	0.18
Proximal colon	194 (72.1)		0.5 (0.2–1.6)	0.25
Degree of differentiation				
Low grade	354 (75.3)	0.12^a^	1	
High grade	85 (82.5)		1.2 (0.4–3.4)	0.70
Histological classification				
Adenocarcinoma	439 (75.7)	0.29^a^		
Mucinous Adenocarcinoma	38 (73.1)			
Signet-ring cell carcinoma	8 (100.0)			
Other carcinomas	3 (100.0)			
Cancer committee				
No	180 (72.6)	0.30^a^		
Yes	282 (76.4)			
Surgeon’s profile				
General	134 (69.4)	0.07^a^	1	
Coloproctologist	323 (76.5)		1.3 (0.6–3.1)	0.50
Curative resection				
R0	428 (76.0)	0.62^a^		
R1/R2	40 (72.7)			

^a^Pearson Chi-square test

^b^Chi-square test for trends

The most frequent chemotherapy schemes were CAPOX (capecitabine, oxaliplatin) in 49.4% of patients, FOLFOX (5- Fluorouracil, oxaliplatin) in 26.9% and capecitabine in monotherapy in 20% of the cases. Oxaliplatin-based adjuvant chemotherapy administration varied with age as follows: 83.4% in the younger group, 64.2% in the older and 29% in the oldest (p, χ^2^_trends_ < 0.0005). The administration of capecitabine in monotherapy was 11.7, 24.6 and 57.9%, respectively, (p, χ^2^_trends_ < 0.0005).

### Preoperative radiotherapy for patients with rectal cancer

**Fig. 3:**
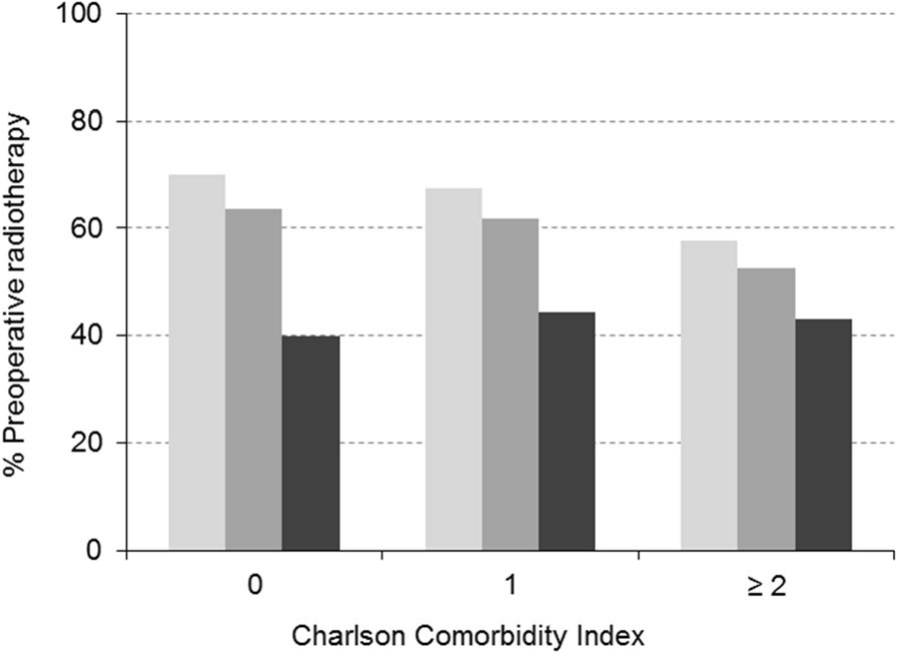
Percentage of patients with stage II and III rectal cancer who received preoperative radiotherapy by age and number of comorbidities. Legends: Age (years)  < 65,  65–80,  > 80

Of the 503 patients with stage II and III rectal cancer, 61% received radiotherapy before surgical intervention. Table 4A shows the univariate association of patient characteristics with preoperative radiotherapy. It was observed that its use decreased significantly with age, from 68% in the youngest age group to 60.4% in the older to 42.2% in the oldest patients (p, χ^2^_trends_ < 0.0005). No significant association was observed between preoperative radiotherapy and sex or with socioeconomic characteristics or living arrangements. We also found significant differences in patients with no comorbidities, with rates of use of 70, 64 and 40% in the three age groups, respectively (p, χ^2^_trends_ = 0.009) (Fig. 3). After simultaneously adjusting for family history of cancer, comorbidities and their severity, and tumour stage (Table 4B), age remained the main predictor. Compared to younger patients, the adjusted OR for the oldest patients was 0.5 (95% CI: 0.3–0.8), while the odds in the group of patients aged 65 to 80 years was not significantly lower with respect to the youngest group. We found no association of CCI or ASA with the use of radiotherapy, but family history was associated with a higher odds of use (OR = 1.5, 95% CI: 1.0–2.2), as was the tumour stage (OR = 2.8, 95% CI: 1.5–4.9).

**Table 4:** Crude and adjusted analysis of the association between age and preoperative radiotherapy in stage II and III rectal cancer patients

	Preoperative radiotherapy in stage II and III rectal cancer			
	4A. Univariate analysis		4B. Multivariate analysis	
	n (%)	*P* value	Odds Ratio (95% CI)	*P* value
Age, years				
< 65	138 (68.0)	< 0.0005^b^	1	
65–80	142 (60.4)		0.9 (0.6–1.4)	0.74
> 80	27 (42.2)		0.5 (0.3–0.8)	0.004
Sex				
Male	205 (60.7)	0.85^a^		
Female	102 (61.8)			
Deprivation index				
Quartile 1	50 (61.7)	0.31^b^		
Quartile 2	86 (54.8)			
Quartile 3	94 (61.8)			
Quartile 4	57 (65.5)			
Level of education				
No formal	31 (62.0)	0.78^b^		
Primary	164 (60.5)			
Secondary	28 (65.1)			
University	25 (55.6)			
Living arrangements				
Living alone	30 (60.0)	0.88^a^		
Living with others	217 (61.3)			
Family history of cancer				
No	147 (55.3)	0.005^a^	1	
Yes	136 (68.3)		1.5 (1.0–2.1)	0.05
Screening				
No	262 (61.6)	0.30^a^		
Yes	29 (53.7)			
Smoking habits				
Never smoker	136 (60.7)	0.31^a^		
Current smoker	56 (68.3)			
Ex-smoker	113 (58.5)			
Alcohol				
No	264 (61.4)	0.77^a^		
Yes	32 (59.3)			
Charlson index				
0	179 (64.6)	0.03^b^	1	
1	74 (60.7)		0.9 (0.6–1.4)	0.77
≥ 2	54 (51.9)		0.9 (0.5–1.7)	0.88
ASA class				
I-II	187 (63.8)	0.12^b^	1	
III	106 (57.6)		0.9 (0.6–1.2)	0.39
IV	7 (50.0)		0.8 (0.3–2.4)	0.68
Degree of differentiation				
Low grade	201 (59.5)	0.76^a^		
High grade	29 (56.9)			
Histological classification				
Adenocarcinoma	268 (60.8)	0.29^a^		
Mucinous adenocarcinoma	13 (52.0)			
Stage				
II	75 (45.7)	< 0.0005^a^	1	
III	232 (68.4)		2.8 (1.5–4.9)	0.001
Cancer committee				
No	84 (57.9)	0.48^a^		
Yes	205 (61.6)			
Surgeon’s profile				
General	91 (61.1)	1.0^a^		
Coloproctologist	201 (61.3)			

^a^Pearson Chi-square test

^b^Chi-square test for trends

## Discussion

### Chemotherapy

In our cohort of patients treated between 2010 and 2012, we found that 70% of all stage III patients with colon cancer received chemotherapy; however, its use dramatically decreased with age, with a percentage of 92% in under-65-year-olds but only 27% among over-80-year-olds. Data from Europe and Australia, where there are health systems with quasi-universal coverage as in Spain, indicate that no more than 20–25% of patients over 75 years old received adjuvant chemotherapy in 2000. In the USA, these percentages reach 40 to 50% [21]. In Spain, on the basis of population data, a study reported that the percentages of chemotherapy use fall from 61% in under-75-year-olds to 27% in patients 75 years of age or older [22].

In our study, a quarter of patients between 65 and 80 years old did not receive any chemotherapy. In some patients, this is attributable to a higher level of comorbidity, but we observed that the pattern remains even in patients with no comorbidities. Moreover, variables such as high alcohol intake, tumour characteristics (site and histological findings), and even curative resection had less influence than age on the decision of whether to treat. This is consistent with previous scientific reviews that have demonstrated a lower use of chemotherapy among the older even after adjusting for comorbidity and other relevant clinical variables [2, 21].

A low level of education, area of residence deprivation and marital status have been reported to be associated with lower probability of treatment [15, 23, 24]. In our study, we have observed that the magnitude of the association between age and chemotherapy does not change when we adjust for level of education, which means that the lower level of education in older patients does not help to explain the differences observed by age group. The deprivation index and living arrangement were also not found to be significantly associated with the use of chemotherapy.

In agreement with previous authors, we observed that those older than 65 were less likely to be treated with chemotherapy in spite of its survival advantage [25, 26]. Furthermore, the very old patients who received chemotherapy were more likely to be treated with capecitabine in monotherapy. Further research needs to be done in the oldest age groups, who have been excluded from most clinical trials and for whom little knowledge on treatment efficacy and safety is available [27].

### Preoperative radiotherapy

The percentages of use of preoperative radiotherapy among patients under 65, between 65 and 80 and over 80 years of age were 68, 60 and 42%, respectively. The decrease with increasing age remained significant after adjusting for comorbidities and the other covariates. Compared to patients under 65 years of age, the adjusted ORs for patients between 65 and 80 and those over 80 years of age were 0.9 and 0.5, respectively.

Previously available evidence, derived from population-level data, indicated less use of radiotherapy among older patients. In Spain, 24% of under-75-year-olds and 11% of patients 75 years of age or older with colorectal cancer have received radiotherapy [22, 28]. In Sweden, the use of preoperative radiotherapy falls from 64% in under-65-year-olds to 15% in over-80-year-olds [7]. According to a review by Faivre [21], the rates of pre- and post-operative radiotherapy ranged from 20 to 50% in different registries in Europe and the USA.

In our study, comorbidity, area of residence deprivation, education and living arrangements did not predict the decision to treat preoperatively with radiotherapy. We did not find studies that analysed the influence of comorbidities. Previous studies have reported living arrangements and marital status to be significant predictors of the use of radiotherapy [7, 15, 29]. We should note that in our study, the percentage of older patients who lived alone was very low (14%). In other countries, the figures reach 35% in people above 65 years old and 50% in those above 80 years of age. This reflects the level of family support, especially from offspring, for widows/widowers in Spain. In Sweden, a study reported an association with income but not with level of education [7].

Another potentially relevant factor is the distance from the tumour to the anal verge, but there is evidence that this factor is not associated with age [8]. We did not study this issue, but some authors have found a strong association between age and the use of radiotherapy regardless of tumour sub-site location [7].

### Limitations

This study has some limitations that should be recognised. We were not able to contact nearly 9% of eligible patients, and we found that these patients were older than the participants; hence, the older patients included may be a biased sample of the older population. If the clinical status of participants was better than that of those excluded, we could be underestimating the real effect of age on the use of cancer treatments. Another selection bias could be associated with the type of centres included in the study, given that most of them were referral hospitals with specialised units.

Regarding comorbidity, it has been suggested that the CCI may not capture comorbidities well, as it does not measure the severity of comorbid conditions [30]. To compensate for this limitation, at least partially, we included ASA class as a proxy for disease severity.

Apart from comorbidity, another factor that could justify a lower use of treatment in older people is a supposedly greater toxicity. There is some evidence suggesting a lack of association between age and toxicity [31] or even a lower incidence of adverse effects in people above 75 years old [32, 33], attributable to dose reduction and the use of less aggressive treatment regimens in this age group. A recent Danish study found that over-70-year-olds with colorectal cancer were treated with single-agent therapy and at a lower initial dosage and that this chemotherapy dose reduction did not have an impact on disease-free survival or cancer-specific mortality; these outcomes were only different in the older patients who received less than half of the full number of cycles (given to other patients) [11]. Nevertheless, other authors have described a higher level of toxicity with age [2, 34]. In the present study, we did not assess adverse events.

A weakness in determining the causes of the low adherence to clinical practice guidelines for older patients is the lack of information concerning the functional status of patients, which might explain treatment decisions. An alteration in the instrumental activities of daily living has been significantly associated with chemotherapy-related toxicity [35]. Further, poor nutritional status has been described as a predictor of a lower tolerance to chemotherapy, and factors such as malnutrition and frailty have been associated with higher mortality in patients with colorectal cancer undergoing palliative chemotherapy [36]. It would be of interest to know whether the 41 patients excluded because of functional limitations received chemo/radio-therapy but poor functional or cognitive status was used as exclusion criterion in the main study. In the case of radiotherapy, another factor that might hinder treatment is difficulty of access to treatment centres [37], although we think that this factor would not have a great impact in our setting, given that when the distance to the hospital is large, public services provide transport to patients who need it.

In our study, we did not take into account variables such as the opinions of doctors and preferences of patients and their relatives. According to some authors, the opinions and attitudes of doctors may explain the low prescription of adjuvant chemotherapy. In particular, older patients are perceived as being less able to tolerate chemotherapy well [38]. Additionally, doctors perceive that a short life expectancy may limit the benefits of chemotherapy, although it has also been shown that chemotherapy does increase the time to recurrence and overall survival in older patients [11]. Some research has provided evidence that doctors may be less likely to offer adjuvant treatments to older patients [39], and in terms of patient preferences, it has been reported that older patients more frequently decline adjuvant therapy, especially if they lack social support [6, 40]. Yellen et al. found that older patients were not less likely to accept chemotherapy than younger patients but that they were less willing to accept a greater level of toxicity in exchange for longer survival [41].

In our health system, the odds of use of both adjuvant chemotherapy for colon cancer and preoperative radiotherapy for rectal cancer decrease dramatically with age. This conclusion can be partially but not completely explained by a higher frequency and severity of comorbidity among older patients. Nevertheless, curative resection, tumour characteristics and social factors such as deprivation, level of education and living arrangements did not help to explain the observed differences in treatment by age. Indeed, after adjusting for all these factors, significant differences between age groups remained. Further research is required to assess the impact of the functional, cognitive and motor status of patients as well as doctors’ knowledge and attitudes and the preferences of patients and their relatives. Some studies have reported the usefulness of including geriatric assessment tools for daily clinical practice, although their application for identifying patients who are good candidates for adjuvant treatments is not clear, and further research is needed to assess the role of these tools in oncological treatment [3, 42].

## Conclusions

The probability of older patients with colorectal cancer receiving adjuvant chemotherapy and preoperative radiotherapy is lower than that of younger patients and many of them are not receiving the treatments recommended by clinical practice guidelines. Differences in comorbidity, tumour characteristics, curative resection, and socioeconomic factors do not explain this lower probability of treatment. Research is needed to identify the role of physical and cognitive functional status, doctors’ attitudes, and preferences of patients and their relatives, in the use of adjuvant therapies.

## Additional file

Additional file 1: **Table S1.** Distribution of variables before and after imputation. (DOCX 33 kb)

## Data Availability

The datasets used and/or analysed during the current study are available from the corresponding author on reasonable request.

## Abbreviations

ASA: American Society of Anesthesiologists
CAPOX: Capecitabine, Oxaliplatin
CCI: Charlson Comorbidity Index
CI: Confidence Interval
FOLFOX: 5-Fluorouracil, Oxaliplatin
OR: Odds Ratio

## References

[1] Chen RC, Royce TJ, Extermann M, Reeve BB (2012). Impact of age and comorbidity on treatment and outcomes in elderly cancer patients. Semin Radiat Oncol.

[2] Hodgson DC, Fuchs CS, Ayanian JZ (2001). Impact of patient and provider characteristics on the treatment and outcomes of colorectal cancer. J Natl Cancer Inst.

[3] Kordatou Z, Kountourakis P, Papamichael D (2014). Treatment of older patients with colorectal cancer: a perspective review. Ther Adv Med Oncol.

[4] Gatta G, Zigon G, Aareleid T, Ardanaz E, Bielska-Lasota M, Galceran J (2010). Patterns of care for European colorectal cancer patients diagnosed 1996-1998: a EUROCARE high resolution study. Acta Oncol.

[5] Murphy CC, Harlan LC, Lund JL, Lynch CF, Geiger AM (2015). Patterns of Colorectal Cancer Care in the United States: 1990–2010. J Natl Cancer Inst.

[6] Lemmens VE, Janssen-Heijnen ML, Verheij CD, Houterman S (2005). Repelaer van Driel OJ, Coebergh JW: co-morbidity leads to altered treatment and worse survival of elderly patients with colorectal cancer. Br J Surg.

[7] Olsson LI, Granstrom F, Glimelius B (2011). Socioeconomic inequalities in the use of radiotherapy for rectal cancer: a nationwide study. Eur J Cancer.

[8] Martling A, Granath F, Cedermark B, Johansson R, Holm T (2009). Gender differences in the treatment of rectal cancer: a population based study. Eur J Surg Oncol.

[9] Eldin NS, Yasui Y, Scarfe A, Winget M (2012). Adherence to treatment guidelines in stage II/III rectal cancer in Alberta. Canada Clin Oncol (R Coll Radiol ).

[10] Kohne C.-H., Folprecht G., Goldberg R. M., Mitry E., Rougier P. (2008). Chemotherapy in Elderly Patients with Colorectal Cancer. The Oncologist.

[11] Lund CM, Nielsen D, Dehlendorff C, Christiansen AB, Ronholt F, Johansen JS (2016). Efficacy and toxicity of adjuvant chemotherapy in elderly patients with colorectal cancer: the ACCORE study. ESMO Open.

[12] Quaglia A, Lillini R, Mamo C, Ivaldi E, Vercelli M (2013). Socio-economic inequalities: a review of methodological issues and the relationships with cancer survival. Crit Rev Oncol Hematol.

[13] Aarts MJ, Lemmens VE, Louwman MW, Kunst AE, Coebergh JW (2010). Socioeconomic status and changing inequalities in colorectal cancer? A review of the associations with risk, treatment and outcome. Eur J Cancer.

[14] Lemmens VE, van Halteren AH, Janssen-Heijnen ML, Vreugdenhil G (2005). Repelaer van Driel OJ, Coebergh JW: adjuvant treatment for elderly patients with stage III colon cancer in the southern Netherlands is affected by socioeconomic status, gender, and comorbidity. Ann Oncol.

[15] Cavalli-Bjorkman N, Lambe M, Eaker S, Sandin F, Glimelius B (2011). Differences according to educational level in the management and survival of colorectal cancer in Sweden. Eur J Cancer.

[16] Cavalli-Bjorkman N, Qvortrup C, Sebjornsen S, Pfeiffer P, Wentzel-Larsen T, Glimelius B (2012). Lower treatment intensity and poorer survival in metastatic colorectal cancer patients who live alone. Br J Cancer.

[17] Quintana JM, Gonzalez N, Anton-Ladislao A, Redondo M, Bare M, de LN F (2016). Colorectal cancer health services research study protocol: the CCR-CARESS observational prospective cohort project. BMC Cancer.

[18] Esnaola S, Aldasoro E, Ruiz R, Audicana C, Perez Y, Calvo M (2006). Desigualdades socioeconómicas en la mortalidad en la Comunidad Autónoma del País Vasco. Gac Sanit.

[19] Charlson ME, Pompei P, Ales KL, MacKenzie CR (1987). A new method of classifying prognostic comorbidity in longitudinal studies: development and validation. J Chronic Dis.

[20] Hightower CE, Riedel BJ, Feig BW, Morris GS, Ensor JE, Woodruff VD (2010). A pilot study evaluating predictors of postoperative outcomes after major abdominal surgery: physiological capacity compared with the ASA physical status classification system. Br J Anaesth.

[21] Faivre J, Lemmens VE, Quipourt V, Bouvier AM (2007). Management and survival of colorectal cancer in the elderly in population-based studies. Eur J Cancer.

[22] Serra-Rexach JA, Jimenez AB, Garcia-Alhambra MA, Pla R, Vidan M, Rodriguez P (2012). Differences in the therapeutic approach to colorectal cancer in young and elderly patients. Oncologist.

[23] Campbell NC, Elliott AM, Sharp L, Ritchie LD, Cassidy J, Little J (2002). Impact of deprivation and rural residence on treatment of colorectal and lung cancer. Br J Cancer.

[24] Carsin AE, Sharp L, Cronin-Fenton DP, Ceilleachair AO, Comber H (2008). Inequity in colorectal cancer treatment and outcomes: a population-based study. Br J Cancer.

[25] Doat S, Thiebaut A, Samson S, Ricordeau P, Guillemot D, Mitry E (2014). Elderly patients with colorectal cancer: treatment modalities and survival in France. National data from the ThInDiT cohort study. Eur J Cancer.

[26] Mitry E, Bouvier AM, Esteve J, Faivre J (2005). Improvement in colorectal cancer survival: a population-based study. Eur J Cancer.

[27] Pallis AG, Papamichael D, Audisio R, Peeters M, Folprecht G, Lacombe D (2010). EORTC elderly task force experts' opinion for the treatment of colon cancer in older patients. Cancer Treat Rev.

[28] De Angelis R, Sant M, Coleman MP, Francisci S, Baili P, Pierannunzio D (2014). Cancer survival in Europe 1999-2007 by country and age: results of EUROCARE--5-a population-based study. Lancet Oncol.

[29] Sacerdote C, Baldi I, Bertetto O, Dicuonzo D, Farina E, Pagano E (2012). Hospital factors and patient characteristics in the treatment of colorectal cancer: a population based study. BMC Public Health.

[30] Schrag D, Cramer LD, Bach PB (2001). Age and adjuvant chemotherapy use after surgery for stage III Colon Cancer. J Natl Cancer Inst.

[31] Fata F, Mirza A, Craig G, Nair S, Law A, Gallagher J (2002). Efficacy and toxicity of adjuvant chemotherapy in elderly patients with colon carcinoma: a 10-year experience of the Geisinger medical center. Cancer.

[32] Kahn KL, Adams JL, Weeks JC, Chrischilles EA, Schrag D, Ayanian JZ (2010). Adjuvant chemotherapy use and adverse events among older patients with stage III colon cancer. JAMA.

[33] Quipourt V, Jooste V, Cottet V, Faivre J, Bouvier AM (2011). Comorbidities alone do not explain the undertreatment of colorectal cancer in older adults: a French population-based study. J Am Geriatr Soc.

[34] Sargent D, Goldberg R, MacDonald J, Labianca R, Haller D, Shepard L. Adjuvant chemotherapy for colon cancer (CC) is beneficial without significantly increased toxicity in elderly patients. Proc ASCO. 2000;19.

[35] Repetto L, Luciani A (2015). Cancer treatment in elderly patients: evidence and clinical research. Recenti Prog Med.

[36] Aaldriks AA, van der Geest LG, Giltay EJ, le Cessie S, Portielje JE, Tanis BC (2013). Frailty and malnutrition predictive of mortality risk in older patients with advanced colorectal cancer receiving chemotherapy. J Geriatr Oncol.

[37] Lin CC, Bruinooge SS, Kirkwood MK, Hershman DL, Jemal A, Guadagnolo BA (2016). Association between geographic access to Cancer care and receipt of radiation therapy for rectal Cancer. Int J Radiat Oncol Biol Phys.

[38] Hakama M, Karjalainen S, Hakulinen T (1989). Outcome-based equity in the treatment of colon cancer patients in Finland. Int J Technol Assess Health Care.

[39] Newcomb PA, Carbone PP (1993). Cancer treatment and age: patient perspectives. J Natl Cancer Inst.

[40] Hoeben KW, van Steenbergen LN, van de Wouw AJ, Rutten HJ, van Spronsen DJ, Janssen-Heijnen ML (2013). Treatment and complications in elderly stage III colon cancer patients in the Netherlands. Ann Oncol.

[41] Yellen SB, Cella DF, Leslie WT (1994). Age and clinical decision making in oncology patients. J Natl Cancer Inst.

[42] Papamichael D, Audisio RA, Glimelius B, de Gramont A, Glynne-Jones R, Haller D (2015). Treatment of colorectal cancer in older patients: International Society of Geriatric Oncology (SIOG) consensus recommendations 2013. Ann Oncol.

