# Supplementary material for: Impact of age on the use of adjuvant treatments in patients undergoing surgery for colorectal cancer: patients with stage III colon or stage II/III rectal cancer
Source: BMC Cancer. 2019 Jul 25;19:735. doi: 10.1186/s12885-019-5910-z (PMC6659283; doi:10.1186/s12885-019-5910-z)
Supplement: Supplementary file 1 — Table S1. Distribution of variables before and after imputation. (DOCX 33 kb) [file 12885_2019_5910_MOESM1_ESM.docx]

Table S1. Distribution of variables before and after imputation.

|  | **Stage III Colon Cancer**  N=654 | | **Stage II/III Rectal Cancer**  N=503 | |
| --- | --- | --- | --- | --- |
|  | **Before imputation** | **After imputation** | **Before imputation** | **After imputation** |
|  | **N (%)** | **N (%)** | **N (%)** | **N (%)** |
| Deprivation index |  |  |  |  |
| Quartile 1 | 133 (21.3) | 133 (20.3) | 81 (17.0) | 81 (16.1) |
| Quartile 2 | 196 (31.4) | 205 (31.3) | 157 (32.9) | 167 (33.2) |
| Quartile 3 | 161 (25.8) | 182 (27.8) | 152 (31.9) | 168 (33.4) |
| Quartile 4 | 134 (21.5) | 134 (20.5) | 87 (18.2) | 87 (17.3) |
| Unknown | 30 |  | 26 |  |
| Level of education |  |  |  |  |
| Illiterate or with no formal education | 71 (13.3) | 205 (31.3) | 50 (12.2) | 178 (35.4) |
| Primary | 335 (62.7) | 304 (46.5) | 271 (66.3) | 203 (40.4) |
| Secondary | 62 (11.6) | 71 (10.9) | 43 (10.5) | 70 (13.9) |
| University | 66 (12.4) | 74 (11.3) | 45 (11.0) | 52 (10.3) |
| Unknown | 120 |  | 94 |  |
| Screening |  |  |  |  |
| No | 510 (82.0) | 542 (82.9) | 425 (88.7) | 449 (89.3) |
| Yes | 112 (18.0) | 112 (17.1) | 54 (11.3) | 54 (10.7) |
| Unknown | 32 |  | 24 |  |
| Alcohol |  |  |  |  |
| No | 529 (86.4) | 571 (87.3) | 430 (88.8) | 449 (89.3) |
| Yes | 83 (13.6) | 83 (12.7) | 54 (11.2) | 54 (10.7) |
| Unknown | 42 |  | 19 |  |
| ASA class |  |  |  |  |
| I-II | 362 (57.2) | 380 (58.1) | 293 (59.7) | 305 (60.6) |
| III | 239 (37.8) | 242 (37.0) | 184 (37.5) | 184 (36.6) |
| IV | 32 (5.1) | 32 (4.9) | 14 (2.9) | 14 (2.8) |
| Unknown | 21 |  | 12 |  |
